# Wireless, Soft Sensors of Skin Hydration with Designs Optimized for Rapid, Accurate Diagnostics of Dermatological Health

**DOI:** 10.1002/adhm.202202021

**Published:** 2022-11-18

**Authors:** Jaeho Shin, Heling Wang, Kyeongha Kwon, Diana Ostojich, Zach Christiansen, Jaime Berkovich, Yoonseok Park, Zhengwei Li, Geumbee Lee, Rania Nasif, Ted S. Chung, Chun‐Ju Su, Jaeman Lim, Hitoki Kubota, Akihiko Ikoma, Yi‐An Lu, Derrick H. Lin, Shuai Xu, Anthony Banks, Jan‐Kai Chang, John A. Rogers

**Affiliations:** ^1^ Querrey‐Simpson Institute for Bioelectronics Northwestern University Evanston IL 60208 USA; ^2^ Laboratory of Flexible Electronics Technology Tsinghua University Beijing 100085 China; ^3^ Institute of Flexible Electronics Technology of THU Jiaxing Zhejiang 314006 China; ^4^ School of Electrical Engineering Korea Advanced Institute of Science and Technology Daejeon 34141 Republic of Korea; ^5^ Wearifi Inc. Evanston IL 60201 USA; ^6^ Department of Materials Science and Engineering Northwestern University Evanston IL 60208 USA; ^7^ Department of Biomedical Engineering Northwestern University Evanston IL 60208 USA; ^8^ Maruho Co., Ltd. Osaka 531‐0071 Japan; ^9^ Department of Dermatology Northwestern University Chicago IL 60611 USA; ^10^ Department of Mechanical Engineering Northwestern University Evanston IL 60208 USA; ^11^ Department of Neurological Surgery Northwestern University Evanston IL 60208 USA; ^12^ Department of Chemistry Northwestern University Evanston IL 60208 USA; ^13^ Department of Chemical Engineering Northwestern University Evanston IL 60208 USA; ^14^ Department of Electrical Engineering and Computer Science Northwestern University Evanston IL 60208 USA

**Keywords:** biomedical devices, diagnostics, flexible electronics, health monitoring, wireless electronics

## Abstract

Accurate measurements of skin hydration are of great interest to dermatological science and clinical practice. This parameter serves as a relevant surrogate of skin barrier function, a key representative benchmark for overall skin health. The skin hydration sensor (SHS) is a soft, skin‐interfaced wireless system that exploits a thermal measurement method, as an alternative to conventional impedance‐based hand‐held probes. This study presents multiple strategies for maximizing the sensitivity and reliability of this previously reported SHS platform. An in‐depth analysis of the thermal physics of the measurement process serves as the basis for structural optimizations of the electronics and the interface to the skin. Additional engineering advances eliminate variabilities associated with manual use of the device and with protocols for the measurement. The cumulative effect is an improvement in sensitivity by 135% and in repeatability by 36% over previously reported results. Pilot trials on more than 200 patients in a dermatology clinic validate the practical utility of the sensor for fast, reliable measurements.

## Graphical abstract

This study reports the skin hydration sensor (SHS), a wireless, flexible, skin‐interfaced multi‐sensor system that enables objective and accurate quantification of skin hydration levels. The streamlined form factors with and simple use protocols offer attractive possibilities results and discussion for monitoring skin health in both clinical and home settings.

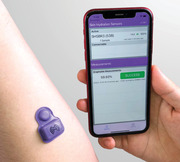

## 1 Introduction

Recent research forms the basis for an increasingly wide range of skin‐interfaced sensors that offer clinical‐grade levels of performance in assessments of health.^[^ ^1^ ^]^ Many such technologies offer wireless capabilities and soft, skin‐compatible mechanical designs to allow routine use not only in hospitals and healthcare facilities but also in the home, for both episodic measurements and continuous monitoring. Of particular interest are sensors that characterize the properties of the skin, the largest organ of the human body, and its three constituent layers: the epidermis, a primary protective structure that includes the stratum corneum; the dermis, a fibrous layer that supports and reinforces the epidermis; and the subcutis, a subcutaneous layer of fatty cells that supplies nutrients to the dermis and epidermis. Of the various essential functions of the skin, its barrier properties are among the most important. Impairments can lead to skin disorders and often precede diseases such as atopic dermatitis (AD) and xerosis cutis (XC).^[^ ^2^ ^]^ Measurements of the trans‐epidermal water loss (TEWL) and the electrical impedance of the skin, performed using probes connected to auxiliary hardware, can yield insights into skin barrier function.^[^ ^3^ ^]^ Alternative approaches rely on thermal transport properties of the skin determined using soft, wireless devices,^[^ ^4^ ^]^ tailored to reveal the water content in the upper layers of skin structure (stratum corneum, SC, and epidermis, ED) through measurements of thermal conductivity.

This paper reports several technical advances that significantly improve the sensitivity and repeatability of such types of thermal sensors. The result is a miniaturized wireless system that integrates multiple sensors based on the transient plane source (TPS) technique. Optimized designs minimize parasitic thermal transport pathways and ensure reliable interfaces to the skin, as examined systematically through a combination of computational and experimental studies. Complementary advances in the measurement protocols minimize variabilities due to human factors, thereby further enhancing the repeatability. Assessments of skin hydration across more than 200 human subjects, with statistical comparisons to results obtained using standard measurement tools and through traditional dermatological scoring systems, illustrate the clinical utility of this technology.

## 2 Results and Discussion

### 2.1 Device Configuration and Working Principles

**Figure 1:**
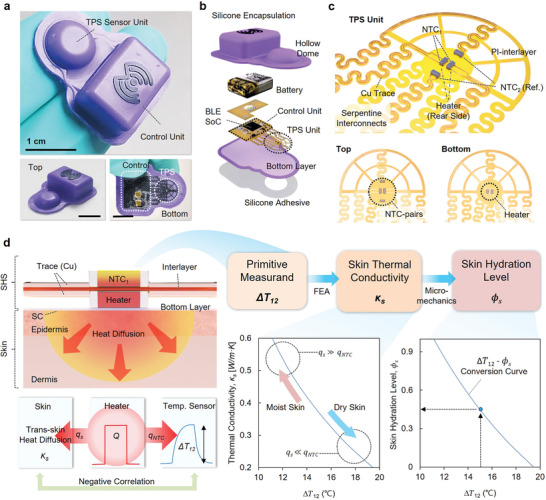
Soft, wireless sensor for noninvasive measurements of skin hydration. a) Images of a skin hydration sensor (SHS), a wireless, flexible system for noninvasive measurements of skin hydration. Scale bars, 1 cm. b) Exploded view schematic illustration of the SHS, highlighting major components: silicone encapsulation with a hollow dome structure, Li‐polymer battery, f‐PCB, fabric‐reinforced silicone bottom layer, and adhesive layer with perforation pattern. c) Highlighted illustration of the TPS unit, highlighting major components: mesh structured polyimide (PI)‐interlayer, Cu traces, serpentine interconnects, NTC_1_, NTC_2_, and heater. Below are the top and bottom views. d) Schematic diagram of the measurement mechanism – two‐step, quantitative conversion of spatiotemporal changes in temperature (Δ ***T***_12_) into the skin hydration level $\phi_{s}$; an FEA model converts Δ ***T***_12_ to skin thermal conductivity (****κ****_***s***_) (left graph), which is sequentially converted to Φ_𝑠_ by a micro‐mechanics model (right graph). Increases in Φ_𝑠_ facilitate thermal transport through the skin, thereby reducing the increase in temperature of the NTC_1_ (Δ ***T***_12_).

**Figure** **1**a,b shows a picture of a device and an illustration of its design, respectively. The sensor, referred to as a skin hydration sensor (SHS), enables TPS measurements with a wireless mode of operation, powered by a Li‐Po battery module. The control electronics include a Bluetooth Low Energy (BLE) system on a chip (SoC) with peripheral components built on a flexible printed circuit board (f‐PCB) laser‐cut into an open architecture with filamentary serpentine interconnects and support features. A user interface (UI) operates on a portable device such as a smartphone. A molded silicone structure with an ultrathin fiberglass‐silicone composite layer at the skin interface encapsulates the electronics and sensor components. The low modulus, flexible mechanical construction allows reliable physical contact and thermal coupling to the skin, even at curved regions of the body (Figure 1b).

Serpentine interconnects join the TPS sensing module to the other parts of the system in a way that mechanically and thermally decouples the two. The TPS module includes a pair of resistive heaters spaced by 160 µm on the bottom side of the f‐PCB, facing toward the skin. On the top side of the f‐PCB, two pairs of temperature sensors (negative temperature coefficient of resistance, NTC, components) reside symmetrically above these heaters (NTC_1_) at a distance of 1 mm (NTC_2_) (Figure 1c). Due to the layout of the device, NTC_2_ depends much more strongly on the temperature of the ambient and less on the temperature of the heaters, compared to NTC_1_. A hollow dome structure of silicone shown in Figure 1b provides thermal isolation. Details appear in Note S1, Supporting Information.

The sensor determines the thermal conductivity of the skin, *κ*_*s*_, which can be converted into the skin hydration level (*ϕ*_*s*_), i.e., the volumetric ratio of water content in the skin, via micro‐mechanics modeling techniques. The SHS measurement begins as the heater generates a constant thermal flux *Q* (27.8 mW in the studies reported here) over a time *t_h_* (3–10 s in the studies reported here). A fraction of this flux passes into the skin (*q_s_*) and the rest into NTC_1_ (*q_NTC_*) such that *q_NTC_* = *Q* − *q_s_*. For identical structural/material sensor configurations, *q_s_* is determined by *κ*_*s*_ and their numerical relation *q_s_* (*κ*_*s*_) can be modeled by finite element analysis (FEA). The remaining flux, *q_NTC_* yields a temperature gradient between NTC_1_ and NTC_2_, as Δ *T*_12_ = Δ*T*_1_ − Δ*T*_2_, where Δ*T*_1_ and Δ*T*_2_ are the changes in temperature of NTC_1_ and NTC_2_ over *t_h_*. This gradient, Δ*T*_12_, does not depend on changes in ambient temperatures as Δ*T*_2_ eliminates these effects on Δ*T*_1_.^[^ ^4^ ^]^ Therefore, FEA can define *q_NTC_* (Δ*T*_12_), which is independent of variations in the ambient. The ideal model in Equation (1) indicates that measurements of *q_NTC_* via Δ*T*_12_ can determine *q_s_*, thus *κ*_*s*_.

(1) $$q_{s}\left(\kappa_{s}\right)=Q-q_{NTC}\left(\Delta T_{12}\right)$$

The value of *κ*_*s*_ can be converted into *ϕ*_*s*_ by micro‐mechanics models, as aforementioned. Figure 1d presents a schematic illustration of the process for extracting *ϕ*_*s*_ from Δ*T*_12_. Detailed descriptions of the measurement principles and analysis approach appear in Note S2, Supporting Information.

### 2.2 Sensitivity Enhancements by Thermal Optimization

**Figure 2:**
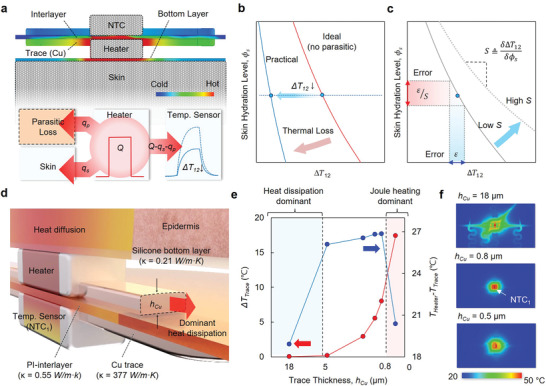
Parasitic mechanisms for heat dissipation and their adverse effects on measurement sensitivity. a) Schematic visualization of parasitic heat dissipation through the interlayer, bottom layer, and Cu trace, the effects of which decrease Δ ***T***_12_. b) Schematic graph exemplifying changes in the dependence of Δ ***T***_12_ on Φ_𝑠_ due to these parasitic heat dissipation mechanisms. c) Schematic explanation of commensurate changes in measurement sensitivity, S, defined as the reciprocal slope of the graph. The parasitic heat dissipation decreases S. d) Illustration of the region near the skin‐sensor interface. The Cu trace represents the major route for heat dissipation. e) Changes in temperature difference between the heater and Cu trace (***T***_**Heater**_ − ***T***_**Trace**_, blue) and the trace temperature (Δ ***T***_**Trace**_, red) as a function of ***h***_***Cu***_. Below a thickness of ≈0.8 µm, Joule heating in the traces becomes significant. f) The top‐view FEA graphics show the temperature distributions for cases of ***h***_***Cu***_= 18 µm (top), 0.8 µm (middle), and 0.5 µm (bottom). The ***h***_***Cu***_ = 0.8 µm configuration shows the most confined temperature distribution with the highest peak temperature. The dimensions are exaggerated for visualization in a and b.

Parasitic thermal pathways within the device structure generate an additional thermal flux *q_p_*, which alters the ideal scenario described above (**Figure** **2**a) such that

(2) $$q_{s}=Q-q_{NTC}-q_{p}=\left(Q-q_{p}\right)-q_{NTC}$$

The effect of *q_p_* reduces *q_NTC_* and, thus, Δ*T*_12_ at any given *ϕ*_*s*_ (Figure 2b). Figure 2c illustrates that the effect of *q_p_* is to reduce the slope of the curve that defines the relationship between Δ*T*_12_ and *ϕ*_*s*_. This reduction corresponds to a decrease in the sensitivity *S*, as defined by

(3) SΔ=δΔT12δϕs

where *δ* denotes changes in these variables.

Reducing *q_p_* thus enhances *S*. The copper ( *κ*_*Cu*_= 377 W m^−1^ K^−1^) electrical interconnects contribute a significant parasitic thermal transport path (Figure 2d). Figure 2e shows FEA results for the changes in temperature of the heater and the interconnects as a function of their thickness (*h_Cu_*) for the case of fixed power at the heater (operating voltage of 3.3 V). The temperature difference between the heater and interconnects (*T*_Heater_−*T*_Trace_, blue line) increases with decreasing *h_Cu_*, as expected. The temperatures for the thick (18 µm) and thin (0.8 µm) cases are different by almost a factor of two (Figure 2f, top, middle). Traces with thicknesses smaller than 0.8 µm can lead to significant Joule heating in the traces themselves (Δ*T_Trace_*, Figure 2e, red line), thereby reducing effective *Q* of the heater in a different manner, but with a similar consequence in a reduced *S* (Figure 2f, bottom).

**Figure 3:**
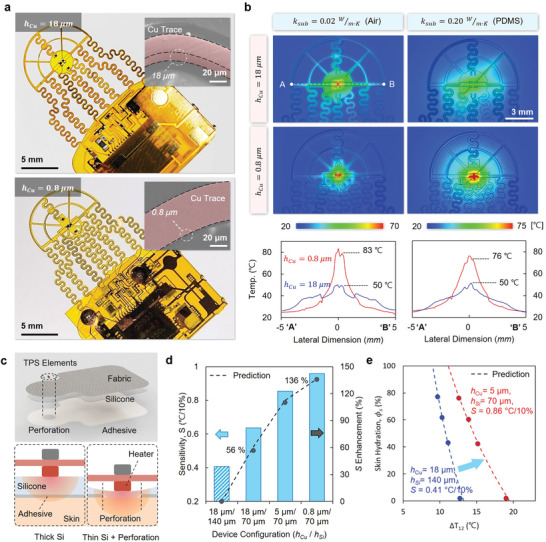
Improvements enabled by thin metal interconnects and simplified skin‐sensor interfaces. a) Images of f‐PCBs with ***h***_***Cu***_ = 18 µm (upper) and ***h***_***Cu***_ = 0.8 µm (lower). The insets are SEM images with Cu traces highlighted (red). b) Top‐view infrared images of each device on two substrates: air (****κ****_***air***_ = 0.02 W m^−1^ K^−1^, left column) and PDMS (****κ****_***PDMS***_ = 0.20 W m^−1^ K^−1^, right column). The graphs below show the temperature profiles of each device along the horizontal line marked with “A” and “B” in the images. The outline layouts are overlapped for visibility. c) Schematic illustrations of the optimized bottom layer structure. Reductions in the thickness of the silicone layer (140 µm → 70 µm) and the adhesive layer perforation (6.5 mm diameter) are highlighted. d) Sensitivity enhancement by device configurations. Compared to the standard device (***h***_***Cu***_ = 18 µm, ***h***_***Si***_= 140 µm), the thin bottom layer increases the sensitivity by 56%, and the use of thin traces results in an additional 80% enhancement. Collectively, the structural optimizations enhance the sensitivity by 136%. e) Graphs of Δ ***T***_12_ as a function of Φ_𝑠_ for the unoptimized and optimized cases. A decreasing slope corresponds to an increase in sensitivity.

Measurements using SHS devices with different values of *h_cu_* experimentally validate these FEA results (**Figure** **3**a). Infrared imaging (Figure 3b) during TPS measurements at the peak temperature of the heater (in air, *κ*_*air*_ *=* 0.02 W m^−1^ K^−1^; on silicone substrate, *κ*_*PDMS*_ *=* 0.20 W m^−1^ K^−1^) reveals a strong reduction in heat dissipation for ultrathin (0.8 µm) Cu traces compared to that of thick, industry‐standard (18 µm) traces. The resulting improved thermal confinement enhances Δ*T*_12_. Temperature profiles indicate a specific enhancement, from point “A” to “B” in the images, of 50%–60% in the peak temperatures. The results, however, do not suggest a similar increase in skin temperature (Δ*T_Skin_*). Due to heat transfer through the bottom layer and the skin, Δ*T_Skin_* remains under 10 °C in actual cases, thereby avoiding any sensation of pain or physical damage to the skin.^[^ ^5^ ^]^ Details appear in Note S3, Supporting Information.

The design of the skin‐sensor interface can also be optimized to enhance *S*. The silicone bottom encapsulation layer and adhesive that bonds the sensor to the skin form the interface. A high thermal interface resistance impedes *q_s_* and weakens the correlation with Δ*T*_12_, thereby decreasing *S*. A silicone‐fabric composite improves the mechanical strength of the encapsulation to allow reductions in thicknesses (*h_Si_*) to 70 µm, along with corresponding reductions in interface resistance. Creating an opening (6.5 mm diameter) in the adhesive at the location of the heater eliminates its contribution to the resistance (Figure 3c). The consequent thermal resistance per area at the interface reduces by 53% from 750 mm^2^K W^−1^ to 350 mm^2^K W^−1^.

Figure 3d shows that compared with a reference device ( *h_Cu_* = 18 µm, *h_Si_* = 140 µm, *S* = 0.41 °C/10%), a version with this optimized skin‐sensor interface ( *h_Cu_* = 18 µm, *h_Si_* = 70 µm) exhibits a 55% enhancement in sensitivity (*S* = 0.64 °C/10%). An additional 80% enhancement follows from the use of the optimized trace thicknesses described above ( *h_Cu_* = 0.8 µm, *h_Si_* = 70 µm), leading to a cumulative 135% enhancement, corresponding to 0.96 °C/10%. The *ϕ*_*s*_‐Δ*T*_12_ curve that defines the connection between these two parameters appears in Figure 3e, where the experimental data are from artificial skin structures with various *ϕ*_*s*_ equivalents. The theoretical predictions (dashed lines) follow from FEA results. Details of the sensitivity enhancement, together with the other attributes, including precision, long‐term stability, and device‐to‐device variation, are in Supporting Information Notes S4, S5, S6, and S7, respectively.

### 2.3 Repeatability Enhancements by Protocol and Mechanics Optimization

Natural physiological responses lead to increases in skin hydration with occlusion and applied pressure.^[^ ^6^ ^]^ (Note S8, Supporting Information) Measurements using the SHS platform can capture the effects. Specifically, the values of *ϕ*_*s*_ increase (roughly 10%) over the first 3 minutes after applying the device, likely due to occlusion, and then stabilize over the next 10 minutes. The *ϕ*_*s*_ returns to its initial state approximately 5 min after removing the device. A negative correlation between the initial hydration level (*ϕ*_*s*0_) and the rise in *ϕ*_*s*_ after 5 min of skin occlusion (Δ*ϕ*_*s*_) for 10 human subjects suggests that the occlusion effect is most significant for dry skin (low *ϕ*_*s*0_), consistent with poor skin barrier function and corresponding high TEWL. Acclimatization of the skin under a still, room‐temperature (20‐ 22 °C) atmosphere prior to on‐skin measurements can improve the accuracy and repeatability.^[^ ^6^, ^7^ ^]^ Decreasing the measurement time (*t_h_*) is also important in minimizing the effects of occlusion.

The effective depth of the measurement is another important consideration. Most variations in skin hydration levels originate in the top layers of the skin, i.e., SC and viable ED,^[^ ^8^ ^]^ where abnormalities in water content closely correlate with pathological skin conditions, such as AD, XC, and ichthyosis.^[^ ^8b,c^ ^]^ The combined thickness of these layers is ≈100 µm,^[^ ^9^ ^]^ but with large variations among individuals and body locations.^[^ ^10^ ^]^ As a result, an effective measurement depth of ≈200 µm is sufficient to capture almost all cases of interest. The optimized skin‐sensor interface increases the rate of diffusion of heat into the skin, to allow these depths to be reached by thermal diffusion in only ≈3 s, setting the lower bound of the measurement time as 3 s. Penetration into the upper parts of the dermis, which may occur in some cases, has no appreciable effect on the measurement because the dermis is known to maintain a nearly constant hydration level independent of pathological skin conditions.^[^ ^11^ ^]^ A derivation of the optimal measurement time appears in Note S9, Supporting Information.

**Figure 4:**
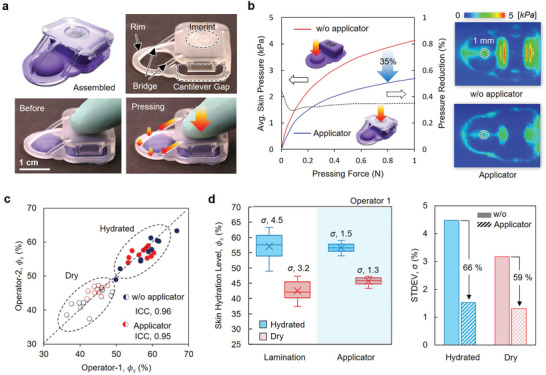
Applicator framework to enhance the repeatability of the measurement. a) Schematic illustration (left upper) and images of the applicator, which incorporates an exoskeletal compliant mechanism. b) The applicator buffers (≈35%) and regulates the pressure manually applied to the skin beneath the TPS unit (blue) compared to the case of applying pressure directly to the TPS dome structure (red). FEA results quantify these reductions in pressure. (right) c) Repeatable, reliable SHS measurements obtained by different users. Results obtained both with and without the applicator show remarkably high intra‐class correlation coefficients (ICC) of 0.95 and 0.96 in repeated measurements on hydrated and dry skin. d) The use of the applicator decreases the variance (standard deviation) of Φ_𝑠_ with repeated measurements by more than a factor of two (66% reduction for hydrated skin, 59% for dry skin).

Changes in *ϕ*_*s*_ that arise from pressure applied by the device to the skin are also important to consider. As with occlusion, the effects of pressure can persist for minutes, likely depending on vascular relaxation and associated vasodilation of nearby arterioles.^[^ ^12^ ^]^ Clinical standard measurement apparatus typically include pressure sensors to guide the application process. Although the SHS does not require applied pressure for the measurement, mechanical manipulation and manual delivery to the skin can result in pressure variations and thus uncertainties in *ϕ*_*s*_. A flexible applicator structure with a compliant architecture^[^ ^13^ ^]^ minimizes these variations by minimizing the contact pressure during use (**Figure** **4**a). The design includes yielding structures (denoted as “bridge” in Figure 4a) that deform slightly to deliver forces evenly to the rim around the sensor region of the device, triggered by manual pressure applied at the back end (denoted as “imprint” in Figure 4a). The result is gentle adhesion of the TPS sensor to the skin for consistent contact before and throughout the measurement. The FEA results in Figure 4b summarize the pressure on the skin near the sensor as a function of applied pressure with (solid blue line) and without (solid red line) the applicator. The findings suggest a 35% reduction in the pressure with the use of the applicator (dashed line), along with improved uniformity in the distributions of strain. SHS measurements from two skin sites (dry and hydrated), performed alternately by two different users with and without the use of an applicator, demonstrate the efficacy of this system. Specifically, the results in Figure 4c show a good correspondence between *ϕ*_*s*_ measured by each user.^[^ ^14^ ^]^ Without the applicator, a broad range of *ϕ*_*s*_ results, likely due to unintended pressure during mounting on the skin. The applicator improves the repeatability by 60% (Figure 4d).

### 2.4 Clinical Validation Studies

**Figure 5:**
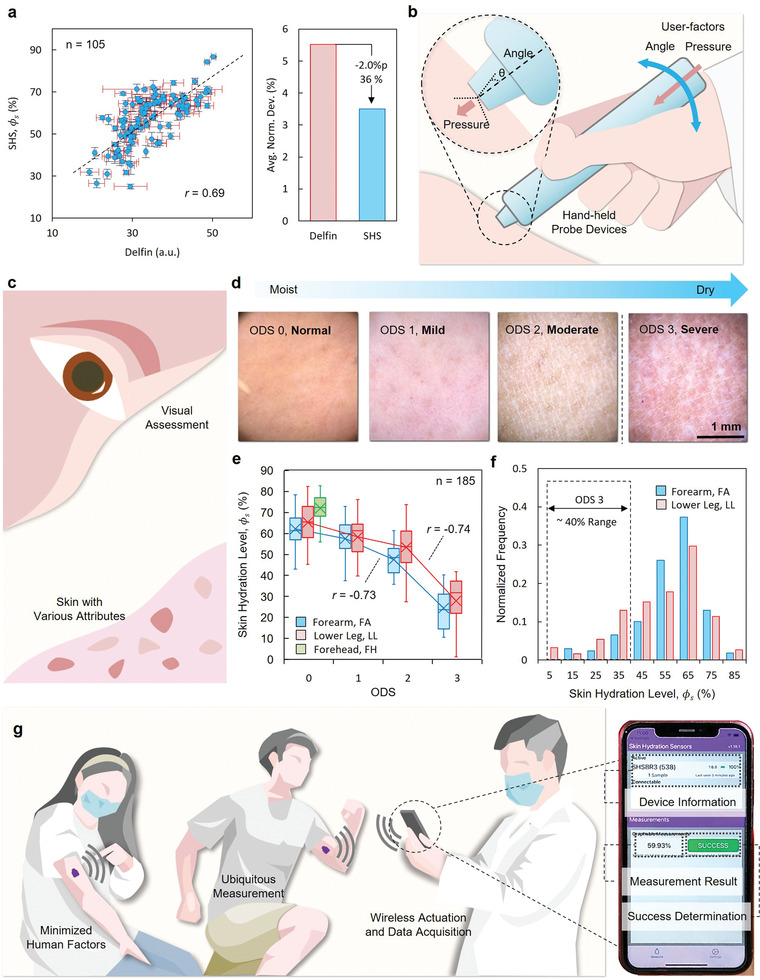
Clinical validation studies. a) Correlation between measurements performed with a commercial tool (Delfin) with an SHS. (*n* = 105, 35 human subjects, triplicate measurements) The Pearson correlation coefficient *r* = 0.69 indicates a strong correlation between the two measures (Left). The SHS results have smaller (by 36%) normalized variances. (Right) b) Illustration of a commercial hand‐held measurement system to highlight human factors that lead to variability. c—f) Comparison with ODS scores. (c) d) Representative images of skin with different ODS. e) Correlation between ODS scores and SHS readings. (*n* = 185, 46 human subjects, 3 skin locations, quadruplicate measurements) The Pearson correlation coefficients *r* = −0.73 and *r* = −0.74 for forearms and lower legs indicate strong correlations between the two measures. f) Normalized distribution of skin hydration levels. Approximately 40% of the hydration spectrum corresponds to ODS3. g) SHS application concept (left) and a digital image of the graphical user interface on a mobile device (right), which includes device information, measurement success determination, and measurement result, Φ_𝑠_.

Clinical pilot studies with 32 elderly patients during routine visits to a dermatology clinic in Chicago, USA demonstrate the practical utility of the technology. The protocols involve triplicate measurements by dermatologists using both the SHS and a commercial system (Delfin), alternately on three skin locations (forehead, lower leg, and lower arm, *n* = 104) for each patient, following an acclimatization period of 20 min. **Figure** **5**a summarizes correlations (*r* = 0.69, with a *p*‐value of 8.9e‐16) and variances between measurements by SHS and Delfin devices at the same skin locations. The data obtained with the Delfin exhibit normalized variances that are more than 30% higher than those recorded with the SHS. These improvements follow from the different operating concepts associated with these two types of devices. Hand‐held probes are susceptible to variations in pressure and contact angle (Figure 5b).^[^ ^15^ ^]^ The soft, skin‐interfaced form factor of the SHS and its wireless operation minimize these confounding effects.

An additional set of studies illustrates the practical application and clinical utility of the SHS through comparisons with clinical conventions for assessing skin dryness based on visual and tactile evaluations (Figure 5c). Overall dry skin scores (ODS), a common means to rank skin dryness on a scale from 0 (healthy normal) to 3 (severely dry; scaly/flaky appearance) according to visual appearance and inspection with a dermatoscope,^[^ ^16^ ^]^ for *n* = 185 elderly patients with dry skin serve as the basis of these studies. The skins with ODS 1 and 2 represent mild/moderate progression of skin dryness where preventive care should be considered (Figure 5d). Patients with ODS 3 demand immediate medical care.^[^ ^16^ ^]^

The results of SHS measurements in quadruplicate and corresponding ODS are shown in Figure 5e. The scatter plot presents the SHS measurement results according to groups defined by ODS for each skin location, showing good agreement between these two different assessments. **Table** **1** summarizes variance (ANOVA) test results among ODS groups. All *p*‐values between different ODS groups are less than 0.001, indicating statistically significant distinctions between SHS measurements against ODS. In particular, the *p*‐value smaller than 0.0001 between ODS 2 and 3 highlights that the SHS measurement provides a reliable means to assess skin dryness for medical decision‐making, thereby bypassing potential subjective factors in the ODS. A 3‐sec rapid measurement time leads to no significant decrease in resolution compared to the 10‐sec case, as mentioned in the previous section.

**Table 1:** *ϕ*_***s***_ ANOVA between ODS classes by measurement time (***t***_***h***_)

ODS comparison		*p*‐value		
		*t_h_* = 10 s	*t_h_* = 5 s	*t_h_* = 3 s
0	1	<0.0001	<0.0001	<0.0001
0	2	<0.0001	<0.0001	<0.0001
0	3	<0.0001	<0.0001	<0.0001
1	2	<0.001	<0.001	<0.001
1	3	<0.0001	<0.0001	<0.0001
2	3	<0.0001	<0.0001	<0.0001

Figure 5f shows distributions of *ϕ*_*s*_ according to skin location, at regions except the forehead, where all the samples belong to ODS 0. In both the forearm and lower leg, the dashed rectangle in the graph highlights that ODS 3 spans over approximately 40% ( *ϕ*_*s*_ = 0 ≈ 40%) of the skin hydration levels. This finding suggests that a wide range of severity levels in skin dryness aggregates into a single indicator (ODS 3). The SHS measurement resolves the severity of skin dryness through a continuous scale, with the ability to monitor progression within each ODS group.

## 3 Conclusion 

This study reports a series of engineering advances that significantly improve the performance of a wireless, soft sensor for skin hydration, and enable its use for accurate, reliable assessments both in clinical and home settings. Specifically, experimental measurements and modeling results define two critical aspects that limit the behaviors of previously reported sensors of this general type. The first involves parasitic heat dissipation through the electronics and thermal resistance at the skin‐sensor interface. Thin, narrow interconnects and simplified skin‐sensor interfaces lead to improvements in sensitivity by 135%. The second follows from natural physiological responses associated with skin occlusion and pressure at the skin interface. The combined use of an improved protocol, i.e., acclimatization periods and rapid measurements, and a skeletal applicator results in a 36% increase in repeatability.

Trials with large numbers of (*n* > 200) patients in a dermatology clinic demonstrate the practical applicability of the technology. The results indicate good agreement between measurements performed with the SHS and with clinical standards based on commercial apparatus and ODS grading. Additional attractive features include a mode of operation that does not involve applied pressure, an ability to gently adhere to soft, curved, and sensitive regions of the skin, and the capacity to perform objective measurements rapidly and accurately. Streamlined protocols, miniaturized form factors, and a user‐friendly BLE wireless interface suggest options for clinical‐grade measurements outside of clinical and laboratory facilities (Figure 5g).

## 4 Experimental Section

### Device Fabrication–Encapsulation

The process for encapsulation began with preparation of the bottom layer by embedding a laser‐patterned fiberglass fabric into a spin‐cast (3000 rpm, 30 s, on a glass slide) layer of silicone (Silbione RTV 4420 A&B, Elkem). A mechanical punch trimmed the silicone‐fabric reinforced composite to define the shape of the bottom encapsulation layer. Uncured silicone (Silbione RTV 4420 A&B) served as an adhesive to bond the f‐PCB with mounted circuit components onto this bottom layer. Casting the same silicone material into a mold defined the top encapsulation structure. Another silicone adhesive (Ecoflex 0030, smooth‐on) bonded the top and bottom structures together along the perimeter to seal the electronics inside. Spin‐casting (8000 rpm, 50 s) another silicone material (Silbione RT Gel 4717 A&B, Elkem) formed a thin adhesive layer on a flat liner surface. A mechanical punch defined a circular opening at the location of the TPS elements. Exposing the bottom encapsulation layer and adhesive surfaces to a corona discharge for 5 s activated the surfaces to allow for permanent bonding between the two. All silicones used here were certified as biocompatible for skin‐interfaced applications.

### Thin Trace f‐PCB

Fabrication of an f‐PCB platform with 0.8 µm‐thick Cu traces followed standard procedures in microfabrication. Consecutive rinses with acetone, isopropyl alcohol, and deionized water, followed by blowing with dry air, cleaned 25 µm‐thick PI films (Kapton HN, DuPont). Photolithography used a negative photoresist (AZ5214, MicroChemicals) spin‐cast onto the cleaned surface of the PI. Exposure to O_2_ plasma (200 W, 5 min) activated the film before electron beam evaporation of 10 nm of Cr as an adhesion promoter and 800 nm of Cu, both at a rate of 0.5 nm s^−1^. Thirty seconds of development (AZ300K, MicroChecmicals) removed the photoresist and patterned the metal by liftoff. Laser machining formed via‐holes at desired locations. Applying a piece of water‐soluble tape (Aquasol) patterned by laser ablation allowed sputter deposition of Cu selectively at the locations of the via‐holes. Rinsing in DI water removed the tape. A laser‐patterning system (ProtoLaser U4, LPKF) defined the cutout design, including the serpentines, in the vicinity of the TPS sensor unit. Low‐temperature soldering using a soldering paste (SDLTLFP10T5, Chip Quik Inc.) enabled low‐temperature, mounting of the circuit components.

### Applicator

A stereolithography (SLA) 3D system (Form 3B, Formlabs) formed the applicator in a flexible resin (Flex 80A, Formlabs). After printing, two cycles of cleaning in IPA (30 s rinse and 5 min soak) and ultrasonication in IPA for 10 min removed the uncured residues. Air‐drying and postcuring with ultraviolet light and heat (10 min, 60 °C, Form Cure, Formlabs) completed the process.

### Imaging and Characterization–FEA Analysis

FEA was performed using a commercial software package (ABAQUS, version 2018). CAD software (Fusion 360, Autodesk) defined the geometries of the device and the skin. The software generated the meshed models based on the four‐node solid element technique. Refined meshes ensured the prediction accuracy. The model determined the distribution of pressure on the skin beneath the TPS unit upon uniform pressure at the press mark of the applicator and upon direct pressure on the top of the dome structure at the TPS unit, both for cases with and without the applicator. The coupling between the device substrate and the skin was achieved via hard contact. The Young's modulus was 6.3 MPa for the applicator (Flexible 80A, Formlabs), 800 kPa for the substrate and the encapsulation of the device (Silbione RTV 4420 A&B, Elkem), and 100 kPa for the skin. All materials were assumed to be incompressible.

### Benchtop Study

Calibration and sensitivity evaluation involved bi‐layered artificial skin models with various effective skin hydration levels formed from two types of silicone materials (Sylgard s170, s184, Dow Corning). The preparation procedures appear in a previous report.^[^ ^4^ ^]^

### Imaging

A scanning electron microscope (Hitachi S‐3400N) yielded the SEM images. An infrared camera (FLIR A645sc) captured the thermographic images. A smartphone camera (Galaxy Note 20, Samsung) collected all of the digital images.

### Human Subject and Clinical Studies

Participants were recruited in the outpatient dermatology clinic of Northwestern Medicine. A total of 188 subjects were recruited, including 18 patients with AD, 139 measurement sites with XC, and 319 healthy normal measurement sites. Measurement sites included the flexor surface of the forearm, the flexor surface of the lower leg, and the midline of the forehead and were exposed to ambiance for acclimatization (>15 min) prior to the measurements. These locations were selected primarily for accessibility with the forehead as an internal control known to have consistently higher hydration compared to other sites. Specific placement of the devices was chosen to avoid subject‐specific factors (e.g., body hair, cuts, tattoos, and makeup). A 5 × 5 cm area at each anatomic site was identified, sanitized with a single‐use alcohol wipe, and marked with a surgical marker to ensure that the same location was tested for all measurements. The study includes objective measurements captured by SHS sensor and Delfin impedance probe with ODS scores derived from a dermoscopic and clinical image across all subjects in the three‐body locations. The ODS scores were reviewed by two dermatologists with the aim of 90% concordance. A third reviewer decided between differing scores. Of note, ODS 4 was not used in this study because the definition for this score is “dominated by large scales, advanced roughness, redness present, eczematous changes, and cracks.”^[^ ^16^ ^]^ Active, open lesions/wounds or cracking of the skin were exclusion criteria. A subgroup of participants was assessed with the SHS sensor before and after moisturizer application (*n* = 68 subjects) and tape stripping of the outermost stratum corneum layer (*n* = 10 subjects). Triplicate measurements were recorded with SHS and Delfin devices at each measurement site. Single‐use alcohol wipes were used to sterilize the devices between measurements. Informed consent was obtained from the human subjects prior to participation in this study. This study was approved by the Northwestern University institutional review board (IRB) (IRB study STU00209010).

## Conflict of Interest

The authors declare no conflict of interest.

## Supporting information

Supporting Information

## Data Availability

The data that support the findings of this study are available in the supplementary material of this article.

## References

[1] a) W. Gao , S. Emaminejad , H. Y. Y. Nyein , S. Challa , K. Chen , A. Peck , H. M. Fahad , H. Ota , H. Shiraki , D. Kiriya , D.‐H. Lien , G. A. Brooks , R. W. Davis , A. Javey , Nature 2016, 529, 509; 26819044 10.1038/nature16521 PMC4996079

[2] a) M. Boguniewicz , D. Y. M. Leung , Immunol Rev 2011, 242, 233; 21682749 10.1111/j.1600-065X.2011.01027.x PMC3122139

[3] a) J. W. Fluhr , K. R. Feingold , P. M. Elias , Exp Dermatol 2006, 15, 483; 16761956 10.1111/j.1600-0625.2006.00437.x

[4] K. Kwon , H. Wang , J. Lim , K. S. Chun , H. Jang , I. Yoo , D. Wu , A. J. Chen , C. G. Gu , L. Lipschultz , J. U. Kim , J. Kim , H. Jeong , H. Luan , Y. Park , C.‐J. Su , Y. Ishida , S. R. Madhvapathy , A. Ikoma , J. W. Kwak , D. S. Yang , A. Banks , S. Xu , Y. Huang , J.‐K. Chang , J. A. Rogers , Proc. NatL. Acad. Sci. USA 2021, 118, e2020398118. 33468630 10.1073/pnas.2020398118 PMC7865173

[5] M. W. Dewhirst , B. L. Viglianti , M. Lora‐Michiels , M. Hanson , P. J. Hoopes , Int J 2003, 19, 267. 10.1080/0265673031000119006 12745972

[6] a) J. d. Plessis , A. Stefaniak , F. Eloff , S. John , T. Agner , T.‐C. Chou , R. Nixon , M. Steiner , A. Franken , I. Kudla , L. Holness , Skin Res Technol 2013, 19, 265; 23331328 10.1111/srt.12037 PMC4522909

[7] E. Berardesca , Skin Res Technol 1997, 3, 126. 27333374 10.1111/j.1600-0846.1997.tb00174.x

[8] a) R. R. Warner , in (Eds.: P. Elsner , E. Berardesca , H. IM ), CRC Press, Boca Raton 1994;

[9] J. Sandby‐Møller , T. Poulsen , H. C. Wulf , Acta Derm Venereol 2003, 83, 410. 14690333 10.1080/00015550310015419

[10] R. F. Rushmer , K. J. Buettner , J. M. Short , G. F. Odland , Science 1966, 154, 343. 5917085 10.1126/science.154.3747.343

[11] D. Barco , A. Giménez‐Arnau , Actas Dermosifiliogr 2008, 99, 671. 19087805

[12] a) R. F. Furchgott , P. M. Vanhoutte , FASEB J. 1989, 3, 27; 2545495

[13] L. L. Howell , Compliant Mechanisms, Wiley‐Interscience, Hoboken 2001.

[14] J. J. Bartko , Psychol Rep 1966, 19, 3. 5942109 10.2466/pr0.1966.19.1.3

[15] J. Pinnagoda , R. A. Tupker , T. Agner , J. Serup , Contact Dermatitis 1990, 22, 164. 2335090 10.1111/j.1600-0536.1990.tb01553.x

[16] J. Serup , Skin Res Technol 1995, 1, 109. 27328437 10.1111/j.1600-0846.1995.tb00029.x

