# Supplementary material for: Wireless, Soft Sensors of Skin Hydration with Designs Optimized for Rapid, Accurate Diagnostics of Dermatological Health
Source: Adv Healthc Mater. 2022 Nov 18;12(4):2202021. doi: 10.1002/adhm.202202021 (PMC11468582; doi:10.1002/adhm.202202021)
Supplement: Supplementary file 1 — Supporting Information [file ADHM-12-2202021-s001.pdf]

Supplementary Information

Wireless, Soft Sensors of Skin Hydration with Designs Optimized for Rapid, Accurate Diagnostics of Dermatological Health

Jaeho Shin[1], Heling Wang[2,3], Kyeongha Kwon[1,4], Diana Ostojich[5], Zach Christiansen[5], Jaime Berkovich[1,6], Yoonseok Park[1], Zhengwei Li[1], Geumbee Lee[1], Rania Nasif[1,7], Ted S. Chung[1], Chun-Ju Su[1], Jaeman Lim[1], Hitoki Kubota[8], Akihiko Ikoma[8], Yi-An Lu[5], Derrick H. Lin[9], Shuai Xu[1,7,9], Anthony Banks[1,5], Jan-Kai Chang[1,5], John A. Rogers[1,6,7,10,11,12,13,14]**

[1] Querrey-Simpson Institute for Bioelectronics, Northwestern University, Evanston, IL 60208, USA
[2] Laboratory of Flexible Electronics Technology, Tsinghua University, Beijing 100085, China
[3] Institute of Flexible Electronics Technology of THU, Jiaxing, Zhejiang 314006, China
[4] School of Electrical Engineering, Korea Advanced Institute of Science and Technology, 34141 Daejeon, Republic of Korea
[5] Wearifi Inc., Evanston, IL 60201
[6] Department of Materials Science and Engineering, Northwestern University, Evanston, IL 60208, USA
[7] Department of Biomedical Engineering, Northwestern University, Evanston, IL 60208, USA
[8] Maruho Co., Ltd., 531-0071 Osaka, Japan
[9] Department of Dermatology, Northwestern University, Chicago, IL 60611, USA
[10] Department of Mechanical Engineering, Northwestern University, Evanston, IL 60208, USA
[11] Department of Neurological Surgery, Northwestern University, Evanston, IL 60208, USA
[12] Department of Chemistry, Northwestern University, Evanston, IL 60208, USA
[13] Department of Chemical Engineering, Northwestern University, Evanston, IL 60208, USA
[14] Department of Electrical Engineering and Computer Science, Northwestern University, Evanston, IL 60208, USA

[*] To whom correspondence should be addressed.
E-mail: jkchang@mywearifi.com, jrogers@northwestern.edu

* None of the material has been published or is under consideration elsewhere, including on the Internet.

■ **Supplementary Note 1: Device Configuration**

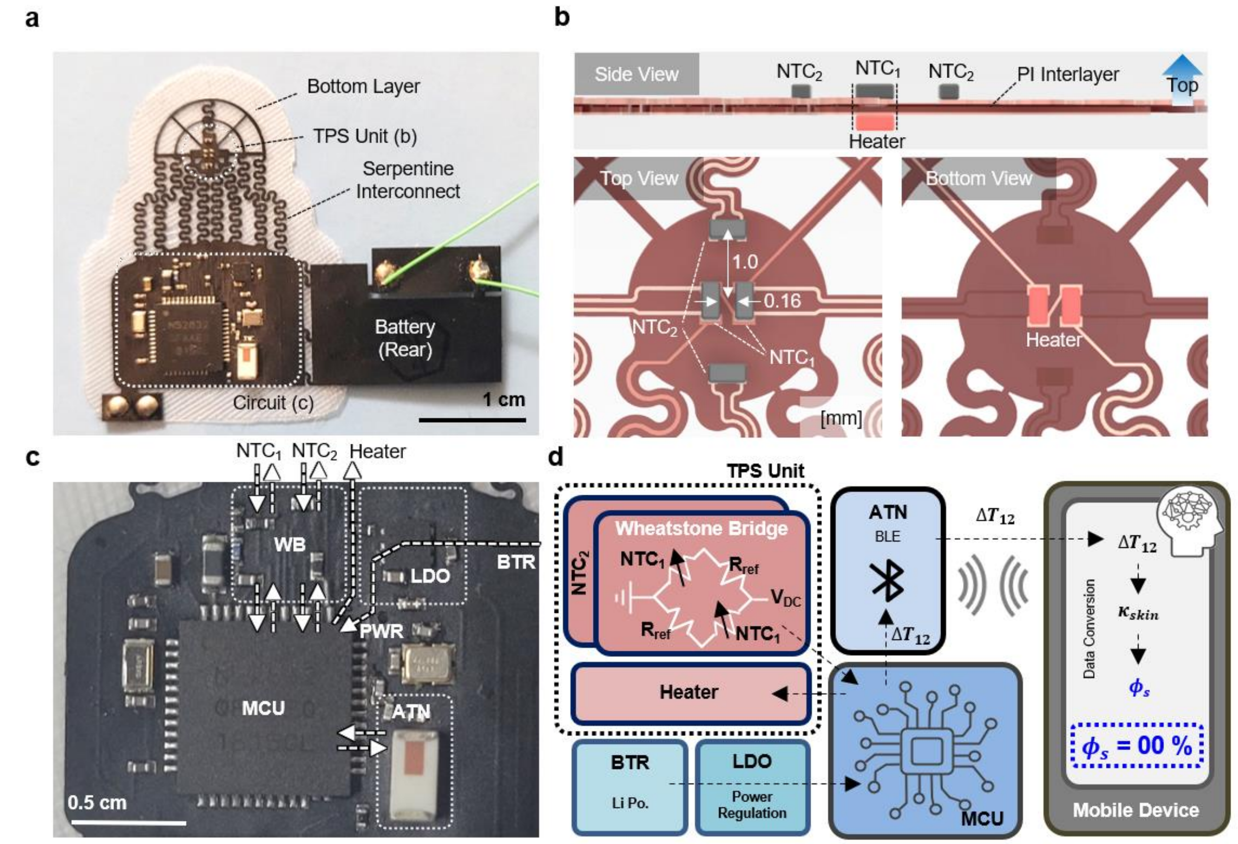

Figure S1. Device configuration. a, Top-view digital image of f-PCB with the bottom layer. The major features, the bottom layer; TPS unit; serpentine interconnect; and circuit, are highlighted. **b**, Highlighted schematic images of the TPS unit. The side view showing NTC_1 and NTC_2 on the topside along with the heater on the other side (Upper). The NTC_1 and heater are aligned symmetrically. Top-view (Lower left) shows the NTCs with relative spacings: 0.16 mm and 1.0 mm. Bottom-view (Lower right) shows the heaters that are series connected. **c**, Magnified digital image of circuit part with augmented marks for major function groups: Wheatstone bridges (WB), low drop out (LDO), microcontroller unit (MCU), and Bluetooth antenna (ATN). **d**, SHS operation architecture. The MCU actuates the heater and digitizes ΔT_{12} from the Wheatstone bridges (NTC_1, NTC_2) to wirelessly transmit to a mobile device through the Bluetooth low energy (BLE) protocol. The mobile device processes ΔT_{12} to elicit ϕ_s.

Figure S1a is a digital image of the f-PCB laminated on the fiber-reinforced bottom encapsulation layer. The TPS unit, consisted of heaters and NTCs, are connected to the control unit by serpentine interconnects. The perforated patterns around the TPS unit are for minimizing thermal

dissipation as well as mechanical stiffness. Figure S1b highlights the TPS units. The side view image shows the vertical alignments among the heaters and NTCs. Two NTCs of NTC_1 pair are facing the heaters for the direct heat transfer with the PI interlayer in the middle. The second NTC pair (NTC_2) are located symmetrically 1 mm away from the center. Figure S1c highlights the controller circuit, which consists of few major sub-function groups. On the upper middle, reference resisters form two sets of Wheatstone bridges along with the two NTC pairs. The Wheatstone bridges enhance the signal to noise ratio. The analogue signals (temperatures) from the Wheatstone bridge circuitries flow into the microcontroller unit (MCU), which functions as the brain of the system. The MCU digitizes the analogue signals and sends them to Bluetooth low-energy (BLE) antenna that enables the wireless communication-ability of the system. The low drop out (LDO) circuitry on the right upper region stabilizes and regulates the battery power to 3.3 V output, which runs the MCU. Figure S1d visualizes these device architectures with block diagrams and pictograms. The SHS transmits ΔT_{12} to an external, typically mobile device. An embedded software converts ΔT_{12} to ϕ_s, applying the conversion methods described in the following section (Supplementary Note 2).

■ Supplementary Note 2: Measurement Principle (Data Conversion)

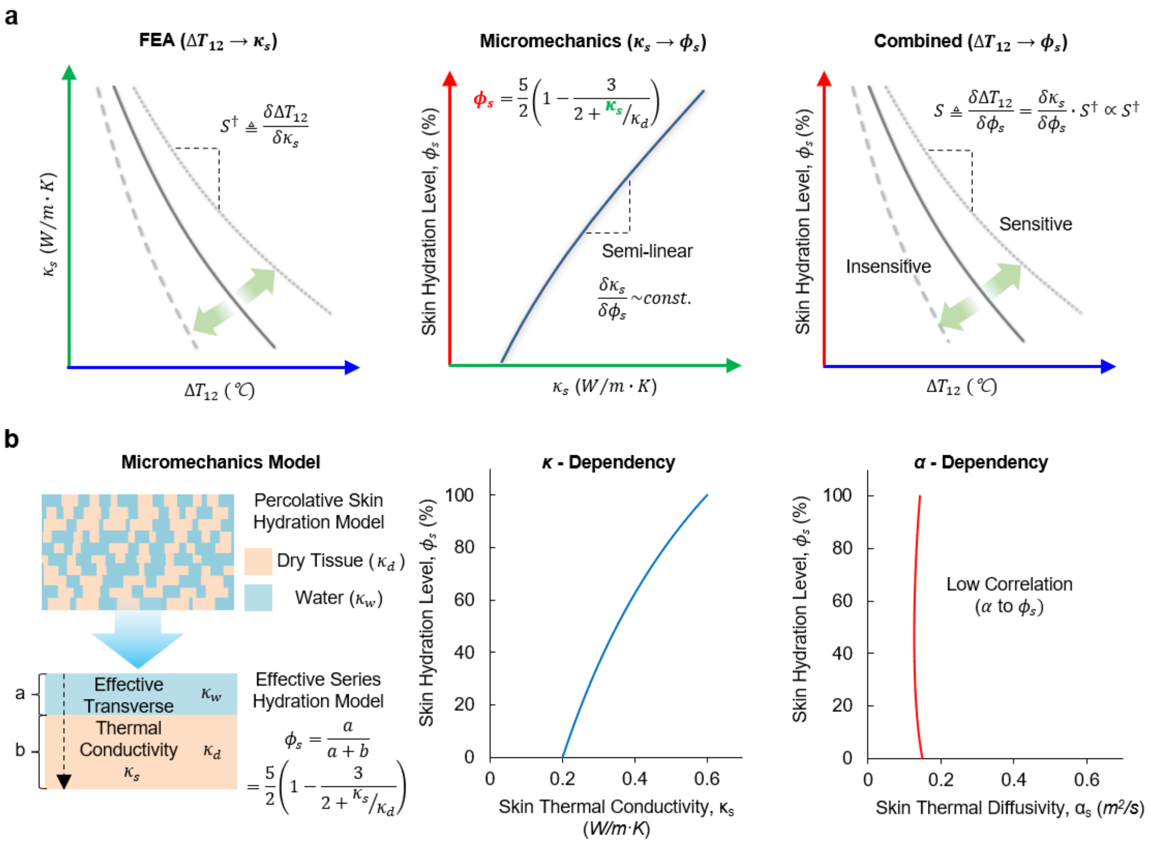

Figure S2. Measurement principle and data conversion. a, Data conversion from ΔT_{12} to ϕ_s. Heat transfer FEA model converts ΔT_{12} to κ_s. The two variables hold a negative correlation with details depending on device configurations. (Left) The micromechanics model relates κ_s to ϕ_s in a semi-linear curve. (Middle) The concatenation of the two conversion models yields the ΔT_{12} to ϕ_s conversion model. The variation in the model (e.g., sensitivity, S) stems from the FEA model. (Right) **b**, Micromechanics model. The model approximates percolative water distribution in the skin to a series connected water-dry tissue bi-layer structure. The effective transverse thermal conductivity κ_s relates to the thermal conductivity of each layer (κ_w, κ_d) and volumetric water content ϕ_s. (Left) The κ_s depends both on the conductivity (κ) and diffusivity (α) of each layer. (Middle, Right) In practice, the diffusivity can be neglected, as the right-most graph indicates.

As can be seen in Figure S2a, SHS elicits ΔT_{12} as a primitive data, which will be converted to ϕ_s later. The conversion is a two-step procedure. The first layer converts ΔT_{12} to κ_s based on an FEA simulation. The simulation elicits a numerical function of ΔT_{12} to κ_s based on the device structure and constituent materials' properties. Thus, the function changes according to the device configurations, such

as constituent materials; geometries; and trace thickness. Each numerical function shows negative correlation between κ_s and ΔT_{12}, since the faster heat transfer into the skin (higher κ_s) implies less heat transferred to the NTC_1 (lower ΔT_{12}).

In the next layer, the micromechanics model converts κ_s to ϕ_s. The model elicits ϕ_s from κ_s, based on the equation below (Equation (S1)) describing the thermal conductivity of a composite material.[1]

$$\frac{\kappa_s}{\kappa_d} = \frac{(p+2) + 2(p-1)\phi_s}{(p+2) - (p-1)\phi_s} \tag{S1}$$

where $p = {}^{\kappa_w}/_{\kappa_d}$, for $\kappa_w = 0.6\, W/m \cdot K$, $\kappa_d = 0.2\, W/m \cdot K$, thermal conductivity of water and dry skin tissue with $\phi_s = 0\%$, respectively.[2] Since the overall thermal transport across the skin will be facilitated as the water contents (ϕ_s) increases, κ_s and ϕ_s show a positive correlation, especially semi-linear. Worth noting is that the micromechanics model does not associate with the device designs.

Combining the two correlations yields the final conversion model connecting ΔT_{12} to ϕ_s. The embedded software described in Figure S1d is based on this conversion model. In this concatenation of the conversion models, an important observation regarding sensitivity (S) can be made.

$$S = \frac{\delta \Delta T_{12}}{\delta \phi_s} = \frac{\delta \Delta T_{12}}{\delta \kappa_s} \cdot \frac{\delta \kappa_s}{\delta \phi_s} \tag{S2}$$

In Equation (S2), the chain rule breaks down the S into two terms from each conversion layer. Since the micromechanics model is invariant to device designs and also the graph is semi-linear, the last term is practically a constant, which gives a contraction of S with reduced sensitivity, $S^{\dagger}$ (Equation (S3)).

$$S \sim S^{\dagger} = \frac{\delta \Delta T_{12}}{\delta \kappa_s}$$

(S3)

The sensitivity of the SHS measurement is entirely dependent on the FEA model, thus, the device configurations.

The left of Figure S2b visualizes how the micromechanics model elicits ϕ_s from κ_s. The model approximates a percolative water distribution inside the skin to a series-connected bi-layer of water and dry skin tissue. The model derives ϕ_s that is required to reconstruct the κ_s from κ_w and κ_d. The rearrangement of Equation S1 gives the following (Equation S4).

$$\phi_s = \frac{p+2}{p-1}\left(1 - \frac{3}{2 + {\kappa_s}/{\kappa_d}}\right) = \frac{5}{2}\left(1 - \frac{3}{2 + {\kappa_s}/{\kappa_d}}\right)$$

(S4)

The last term reflects $p = {\kappa_w}/{\kappa_d} = 3$.

The heat transfer inside the skin relies on not only thermal conductivities but also thermal diffusivities. However, as can be seen on the right-hand side of Figure S2b, theoretical expectation on the skin thermal diffusivity virtually has no change over the full spectrum of ϕ_s. Therefore, the conversion does not necessarily require the consideration of thermal diffusivity.

■ **Supplementary Note 3: Skin Thermal Safety Concerns**

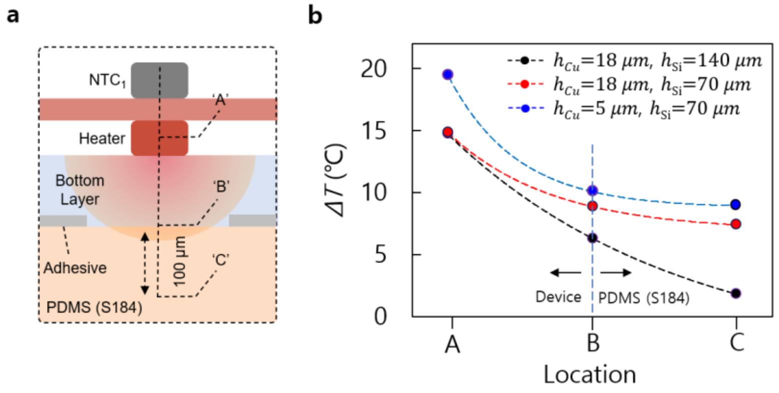

Figure S3. a. Schematic illustration of the experimental setup. **b**. Temperatures at different locations through the thickness of the structure for different trace (h_{Cu}) and bottom layer (h_{Si}) thicknesses. Symbols and dashed lines are measurements and simulation results, respectively.

Experimental and simulation results of the actual use environment suggest that the temperature rise at the skin surface (ΔT_{Skin}) remains under 10 °C in all cases. For heating times of shorter than 10 s, this complies with ISO 13732-1 for ergonomic considerations in contact with hot surfaces.

The experiments used sensors with different trace (18 μm, 5 μm) and bottom layer (140 μm, 70 μm) thicknesses for measurements on two PDMS (Sylgard s184, $\phi_s = 0\%$) substrates. NTCs embedded in each PDMS substrate at the surface and at a depth of 100 μm, respectively, allowed for measurements of temperature at these locations. The optimized bottom layer ($h_{Si} = 70\ \mu m$, an opening in the adhesive layer below the heater, Figure 3c) was applied. (Figure S3a) The experiments involved actuation of the heater for 10 s ($t_h = 10\ s$).

Figure S3b displays the maximum temperature rise associated with activation of the heater. The symbols and dashed lines correspond to the experimental data and simulation results, respectively. Considering that the PDMS substrate corresponds to an extremely dry skin ($\phi_s = 0\%$), the actual skin temperature rise is expected to remain under 10 °C. In addition, in the case of the optimal heater actuation

($t_h = 3\ s$), this expectation will be even lower. The conclusion is that the maximum skin temperature rise remains below 10 °C. Furthermore, the skin will experience the top temperature only in a later portion of the heating period. The tolerance of the skin to such heat budget can be assessed by examining the isoeffect curve associated with thermal damage to human skin.[3] The combination of duration and extent (shorter than 3 s and lower than 10 °C) corresponds neither to pain sensations nor actual physical damage,[3] suggesting the thermal safety of the skin hydration sensor.

■ **Supplementary Note 4: Sensitivity Degradation and Enhancement**

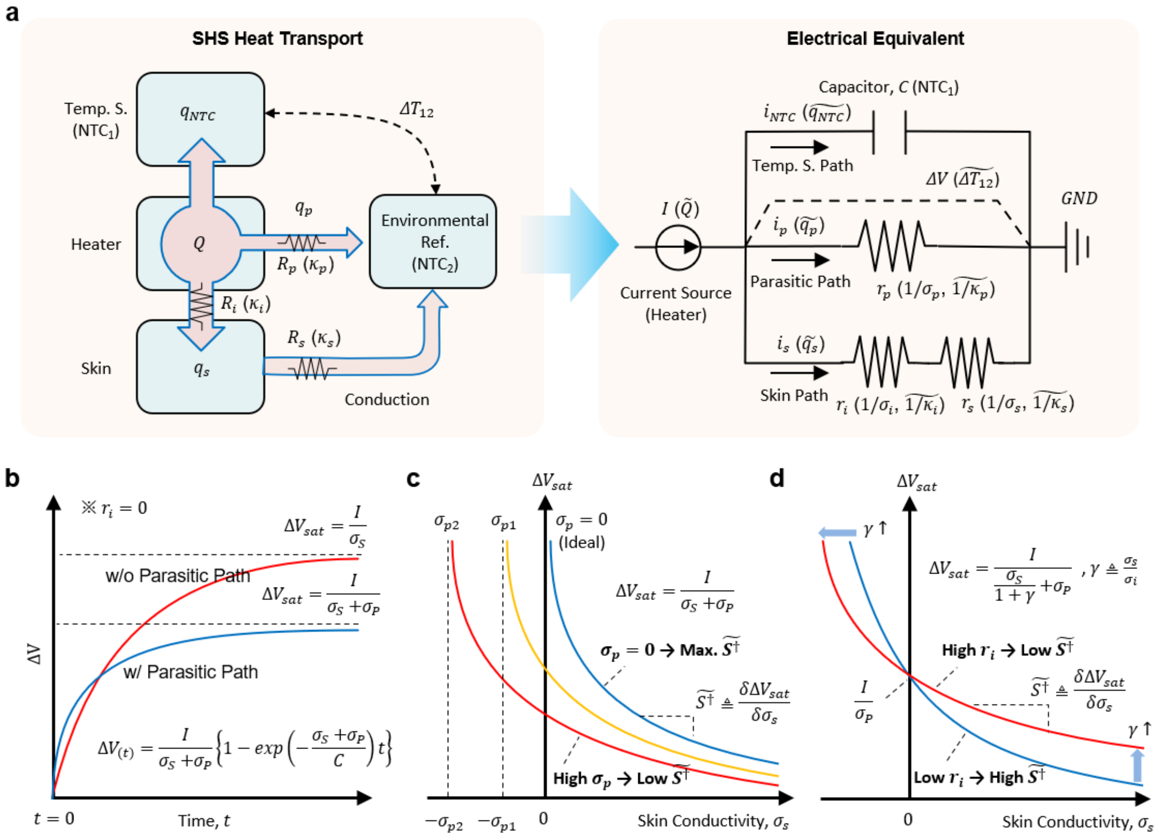

Figure S4. Sensitivity degradation mechanisms. a, Schematic SHS heat transfer structure (Left) and an equivalent electrical circuit (Right). The corresponding thermal variables are denoted with a wave hat notation. **b**, Schematic time-dependent evolution of ΔV, the correspondence of ΔT_{12} according to the electrical conductivity of the parasitic path (σ_p). Interfacial resistance (r_i) is assumed to be 0 without losing the generality. **c**, The ΔV_{sat} as a function of σ_s ($\widetilde{\kappa_s}$), corresponding to σ_p ($\widetilde{\kappa_p}$). ΔV_{sat} is a correspondent of ΔT_{12}, thus the slope of the graph ($\widetilde{S^{\dagger}}$) represents $S^{\dagger}$, the surrogate of sensitivity S. Change of graphs visualizes the measurement sensitivity (S) degradation as the σ_p increases. **d**, The graphical change according to the interfacial resistance, r_i (R_i). The γ indicates the ratio of σ_s to σ_i. As γ increases, $\widetilde{S^{\dagger}}$ decreases, meaning the S reduction due to thermal resistance at the sensor-skin interface.

Figure S4a shows a simplified schematic of the SHS heat transfer structure (Left). The heat (Q) generated at the heater diffuses through three pathways, which are NTC_1, inter-skin, and parasitic paths, following the temperature gradient between the heater and the environment (NTC_2). The right-hand side is an equivalent electrical circuit,[4] consisting of a capacitor and two parallel conduction paths with

proper resistances. The correspondent original variables are denoted in the parentheses with the wave hat notation. The heater, NTC_1, and NTC_2 correspond to the current source, capacitor, and ground, respectively.

The solution of the electrical equivalent circuit gives a time evolution of ΔV, the correspondent of ΔT_{12} as below (Equation (S5))

$$\Delta V_{(t)} = \frac{I}{\left(\sigma_i^{-1} + \sigma_S^{-1}\right)^{-1} + \sigma_P}\left\{1 - exp\left(-\frac{\sigma_S+\sigma_P}{C}\right)t\right\}$$

(S5)

Where dimensionless electrical conductivities σs are correspondents of thermal conductivities, κs; dimensionless capacitance C is correspondents of the thermal capacity of NTC_1. Figure S4b shows the behaviors of ΔV over time, with and without σ_p, in the case of no interfacial resistance r_i. The apparent difference between the two cases is the ΔV saturation level, ΔV_{sat}; the parasitic conduction path decreases the ΔV_{sat}. This ΔV_{sat} represents saturation ΔT_{12-sat}, which is a good approximation of ΔT_{12}, the measurand to elicit ϕ_s.

Figure S4c illustrates the graphs of ΔV_{sat} to σ_s, skin conductivity, with various σ_p. The key feature is the decreasing slope of the graphs following increasing σ_p. The slope of the graphs ($\widetilde{S^\dagger}$) is the correspondent of the reduced sensitivity $S^\dagger$, defined in the previous section and a good benchmark of measurement sensitivity S.

$$\widetilde{S^\dagger} = \frac{\delta V_{sat}}{\delta \sigma_s} = \frac{\widetilde{\delta \Delta T_{12}}}{\delta \kappa_s}$$

(S6)

Therefore, decreasing $\widetilde{S^\dagger}$ with increasing σ_p indicates a decrease in sensitivity as κ_p increases; higher heat dissipation through the parasitic path degrades the measurement sensitivity.

When it comes to the interfacial resistance, we may introduce $\gamma = {}^{\sigma_s}/_{\sigma_i}$ to simplify ΔV_{sat}. (Equation (S7))

$$\Delta V_{sat} = \frac{I}{\left(\sigma_i^{-1} + \sigma_s^{-1}\right)^{-1} + \sigma_P} = \frac{I}{{}^{\sigma_s}/_{1+\gamma} + \sigma_P} \tag{S7}$$

As shown in Figure S4d, the graph of ΔV_{sat} to σ_s becomes flattened with higher γ, meaning the higher the skin-sensor interfacial thermal resistance, the smaller the sensitivity S.

In summary, parasitic heat transfer and sensor-skin thermal resistance deteriorate the measurement sensitivity of SHS. This result sets the rationale for the strategies for sensitivity enhancement introduced in the main manuscript.

■ **Supplementary Note 5: Measurement Precision**

The accuracy of a sensor is an important measure of its performance. The assessment of the accuracy depends on comparisons to the ground truth. In case of the skin hydration sensor, although absolute reference measurements of skin hydration level do not exist, several probe-type systems and the ODS approach serve as *de facto* clinical standards for semi-quantitative surrogates that can be related, roughly, to volumetric water fractions in the skin. Results from our system can be calibrated to these *de facto* standards.

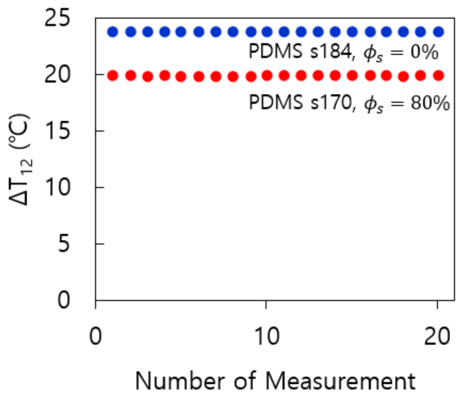

Figure S5. Change of ΔT_{12} over 20 successive measurements on two reference silicone substrates. The ΔT_{12} average deviation is 0.06%, which corresponds to ±0.07% ϕ_s deviation.

Precision, or variations associated with multiple cycles of identical measurement operations, is much easier to define, thus it becomes a good measure for the quality of skin hydarion sensor measurement. Figure S5 displays the changes in ΔT_{12} over 20 successive measurements on two reference substrates, which correspond $\phi_s = 0.0\%$ (blue) and $\phi_s = 80.0\%$ (red), respectively. The measurements used a skin hydration sensor ($h_{Cu} = 5\ \mu m$, $h_{Si} = 70\ \mu m$) without calibration. The heating time was set to 10 s. The intrinsic deviations of ΔT_{12} are 0.04% and 0.07% for each substrate, or 0.055% on average. This range of deviation likely arises from intrinsic variabilitie sin components (heaters, NTCs), solder

joints and other aspects associated with manufacturing. Given sensitivity of $S = 0.86\,℃/10\%\,HL$ (Figure 3d), from calibrated results, the commensurate deviation in the hydration level is ± 0.07% HL. In contrast, Skicon™, a commercialized impedance-based skin hydration level measurement system, reports deviations of ± 10% in microsiemens (μS).[5]

Additional sources of variability arise from physiological processes of the skin, such as those associated with occlusion of trans-epidermal water loss from the and those induced by pressure of the sensor against the skin. The measurement precision in the real environment that reflects all these factors can be seen in Figure 5a. The skin hydration sensor shows 36% lower average normalized deviation over the conventional, hand-held system which relies on a measurement of electrical impedance of the skin. This corresponds to 2.44 times $(1/(1-0.36)^2)$ higher precision of the skin hydration sensor compared to the hand-held system.

■ Supplementary Note 6: Sensor Long-term Stability

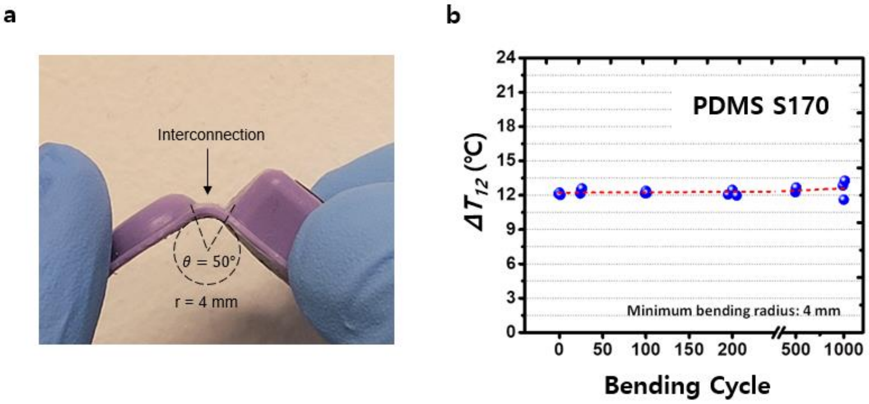

Figure S6. a. The bending of the interconnection of the device. Bending radius of 4 mm. **b**. Change of ΔT_{12} over the cyclic bending test. No appreciable change in ΔT_{12} until 1000 cycles of bending.

Reproducibility of a sensor over a long-term use is another important factor. Because the skin hydration sensor does not incorporate any moving parts, little or no variation in performance for long-term measurements can be expected. To examine the durability and reproducibility of the sensor, cyclic bending tests (to bending radii of 4 mm, much smaller than expected in routine use) were conducted on the mechanically flexible component of the system (Figure S6a). Three measurements were made on a PDMS substrate (Sylgard s170, Dow Corning) just before the 0, 20, 100, 200, 500, and 1000th cycles. As in Figure S6b, the ΔT_{12} shows a stable trend until before 1000 cycles, validating the excellent durability of the skin hydration sensor. At the 1000th cycle, the variation increased to approximately 17%. For more typical bending radii of >1 cm, even better stability performance can be expected. In the clinical validation studies across more than 200 human subjects using more than 20 devices, no signs of drift or instability were observed.

■ **Supplementary Note 7: Sensor Device-to-Device Variation (Calibration Protocol)**

There are slight device-to-device differences due to intrinsic variabilities in components (heaters, NTCs), solder joints and other aspects associated with manufacturing. The devices were, however, each calibrated before deployment to eliminate effects of these variations. The calibration protocols appear below.

Calibration Protocol ---

The devices are mounted on two reference PDMS substrates with different thermal conductivities: Sylgard s184 and Sylgard s170 (Dow Corning). The reference substrates are sufficiently thick to eliminate any effect of the supports. The thermal conductivity of each substrate is given as follows.

- Sylgard s184: $\kappa_{PDMS184} = 0.20\ W/m \cdot K$ / - Sylgard s170: $\kappa_{PDMS170} = 0.48\ W/m \cdot K$

These thermal conductivities correspond approximately to skin with hydration levels of 0.0% and 80.0%, respectively, by the micromechanics model formulated by Equation S1. (Supplementary Note 2)

$$\frac{\kappa_s}{\kappa_d} = \frac{(p+2) + 2(p-1)\phi_s}{(p+2) - (p-1)\phi_s} \qquad \text{(S1)}$$

where $p = \kappa_w/\kappa_d$, for $\kappa_w = 0.6\ W/m \cdot K$, $\kappa_d = 0.2\ W/m \cdot K$, thermal conductivity of water and dry skin tissue with $\phi_s = 0\%$, respectively.

Five sequential measurements on each reference substrate yielded average results. Comparisons to reference values (0% and 80%) determined a linear correction model for each device. The correction was embedded in the firmware of each device before the deployment. However, a sensitivity of $0.5\ ℃/10\%$ serves as a threshold for rework since this calibration does not refine the sensitivity. The failure to meet the threshold is rare, but removing excessive solder resolves most cases.

This calibration protocol was conducted to two decimal places in percentage, thus the calibration zeros the device theoretically with ± 0.005% variation. Also, as shown in Figure S6b, the high level of reliability of these sensors was confirmed for at least 1000 cycles of use, suggesting the similar level of sensor-to-sensor variabilities can be expected to be maintained among the sensors throughout their uses. In contrast, Skicon™, a commercial impedance-based skin hydration level measurement system, tolerates ± 10% variation for its calibration before every use.[5] Other sources of variability follow from effects of pressure applied to the skin; quality of the skin-device contact and extent of the skin occlusion. The applicator minimizes these effects. Related effects in conventional, hand-held devices are pronounced due to user-related variabilities in application to the skin.

■ **Supplementary Note 8: Skin Responses (Occlusion and Pressure Effects)**

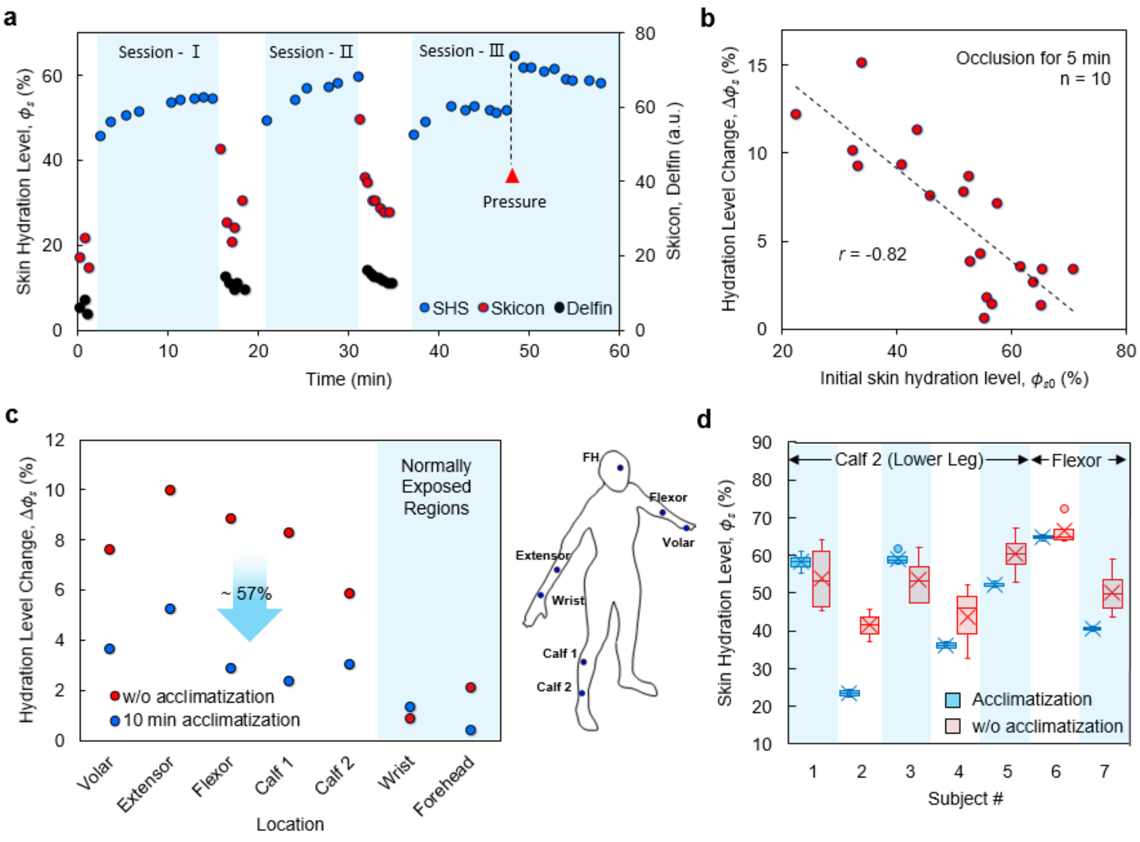

Figure S7. Occlusion effect and the mitigation strategy thereof. a, Consecutive skin hydration level measurements. A skin location on the forearm is examined with SHS as well as two conventional probe-type measurement systems (Skicon^{TM}, Delfin^{TM}). In the first section (section-I), the SHS remains at the location over 9 consecutive measurements, and, in the second section (section-II), SHS and a dummy bottom layer are alternately mounted at the same location. In the third section (section-III), a sudden pressure is applied to the skin location during the consecutive measurement. Between each section, conventional systems alternately monitor the same location. **b**, Negative correlation (r = -0.82) between initial skin hydration level, ϕ_{s0}, and increase according to 5 minutes of skin occlusion, $\Delta\phi_s$. **c**,**d**, The mitigation of the occlusion effect by acclimatization. 10 minutes of acclimatization of the skin mitigates the manifestation of the occlusion effect by approximately half (57% on average) except in regions that are always exposed to the atmosphere (wrist and forehead). (**c**) The acclimatization also reduces variations over consecutive measurements, implying enhanced measurement consistency. (**d**)

Figure S7a shows a series of measurements performed by different protocols and in a dermatology clinic. Each study session involves multiple cycles of measurements using an SHS device

and two different commercial tools (Delfin™; Skicon™) on the same skin location (forearm) of a healthy human subject. Consecutive SHS measurements without removing the device from the skin (denoted as session I) reveals the effect of occlusion of water loss from the skin by the presence of the device. Values of ϕ_s measured by the SHS device rise (roughly 10%) over the first 3 minutes, likely due to occlusion, and then stabilize over the next 10 minutes. The occluded skin tissue can be returned to its initial state by removing the device to expose the skin to air for 5 minutes. During this period, ϕ_s decreases from the elevated to the original level. Similar effects (denoted as study session II) follow from application of a bare silicone layer in the shape of a device on the skin. In Figure S7b, a negative correlation between the initial hydration level (ϕ_{s0}) and the rise in ϕ_s after 5 minutes of skin occlusion ($\Delta\phi_s$) for 10 human subjects shows that the rise is most significant for dry skin (low ϕ_{s0}), corresponding to poor skin barrier function and corresponding high TEWL. These observations provide additional confirmation that the sensor and associated electronics are not responsible for the observed changes. The major consequence is in overestimating the ϕ_s during long measurement periods without device removal.

Contact pressure is another factor that influences measurements of ϕ_s. Figure S7a (session III) shows a rapid increase in ϕ_s increase that follows from application of moderate pressure to the skin during an SHS measurement after the rise and saturation of ϕ_s by skin occlusion for 10 min. Such pressure-induced changes in ϕ_s can persist for minutes. Previous reports suggest that pressure-induced shear stresses on vascular endothelial cells can lead to an increase in local hypoxia. Resulting stimulation and production of vasoactive compounds, including endothelial-derived relaxation factor (EDRF), can then trigger the vasodilation of nearby arterioles, which, in turn, increases the local blood flow and ϕ_s.[6]

The unavoidable presence of these effects of occlusion and pressure demand careful attention to the process of mounting, handling, and applying the devices onto the skin to avoid confounding physiological responses. One of the effective solutions to the uncertainty in measurement practices is to acclimatize the skin to the ambiance before measurement. Figure S7c shows the changes in measured

hydration level ($\Delta\phi_s$) after 5 minutes of skin occlusion at various skin locations of human subjects, with and without a 10 min acclimatization where the human subjects stayed static with their skin exposed to a controlled environment (22 ℃, RH 35%) before device mounting and the subsequent measurements. The acclimatization process reduces $\Delta\phi_s$ by 50% for most of the measured body location, likely due to a relatively stabilized TEWL rate and other reduced factors associated with active skin physiology for the acclimated skin. For measurement on the wrist and forehead, the insignificant changes in $\Delta\phi_s$ after acclimatization can be attributed to their less occluded nature, as the skin of those body locations is usually exposed to the ambience at most of the time. An additional set of clinical trials involving 8 human subjects yields similar results that demonstrate the improved consistency in measuring skin hydration with acclimated skin (Figure S7d), consistent with the clinical practices that a step of acclimatization before a series of consecutive measurements is required to prevent occlusion for assessing hydration across a broad range of skin conditions.

■ **Supplementary Note 9: Optimal Measurement Time (Heater Actuation Time)**

As noted in the main text, the optimized bottom layer enables a fast rate of trans-membrane heat transfer. As a result, the heat transfer enters a quasi-steady state approximately 3 seconds after the heater activation regardless of sub structures. Figure S8 shows that ΔT_{12} reaches ~90% of the saturation level at t = 3s for both cases of PDMS s184 and s170 substrates.

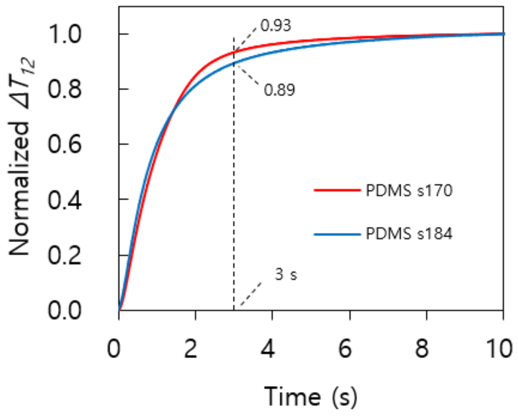

Figure S8. ΔT_{12} saturation of thin bottom layer ($h_{Si} = 70\ \mu m$) devices. ΔT_{12} saturates 3 s after the heater activation on both reference PDMS substrates.

Based on this result, it is possible to estimate the heat penetration depth, as a measure of the depth/range of the TPS measurement. Additional experiments involve a skin phantom structure that consists of a thin layer of PDMS s184 ($\phi_s = 0.0\%$) laminated on a substrate of PDMS s170 ($\phi_s = 80.0\%$). This structure corresponds approximately to the stratum corneum (SC) and epidermis, which are known to be dry and hydrated, respectively. A skin hydration sensor ($h_{Cu} = 5\ \mu m,\ h_{Si} = 70\ \mu m$) performed multiple measurements on the skin phantoms with various thicknesses of PDMS s184 (d_{184}) and a measurement time of 3 s ($t_h = 3\ s$). Figure S9a exhibits data (dots) and the fitting curves (dashed line). Then, the fitting curve is converted to an effective thermal conductivity (κ_{eff}) using Equation S1. (Supplementary Note 2)

$$\frac{\kappa_s}{\kappa_d} = \frac{(p+2) + 2(p-1)\phi_s}{(p+2) - (p-1)\phi_s}$$

(S1)

where $p = \kappa_w/\kappa_d$, for $\kappa_w = 0.6\, W/m \cdot K$, $\kappa_d = 0.2\, W/m \cdot K$, thermal conductivity of water and dry skin tissue with $\phi_s = 0\%$, respectively. Figure S9b shows the effective thermal conductivity curve converted from the hydration level. The converted values indicate the effective thermal conductivity of the skin phantom structure. The conversion returns an effective thermal conductivity of 0.42 W/m·K for d_{184} = 20 μm. This condition reflects the average thickness of SC. Therefore, 0.42 W/m·K can be regarded as an estimation of the effective thermal conductivity for average skin. Figure S9c depicts the heat penetration into the skin phantom structure. In this view, the heat from the heater penetrates through the PDMS s184 layer and reaches to the depth d_{170} into the PDMS s170, corresponding to heat reaching the upper epidermis.

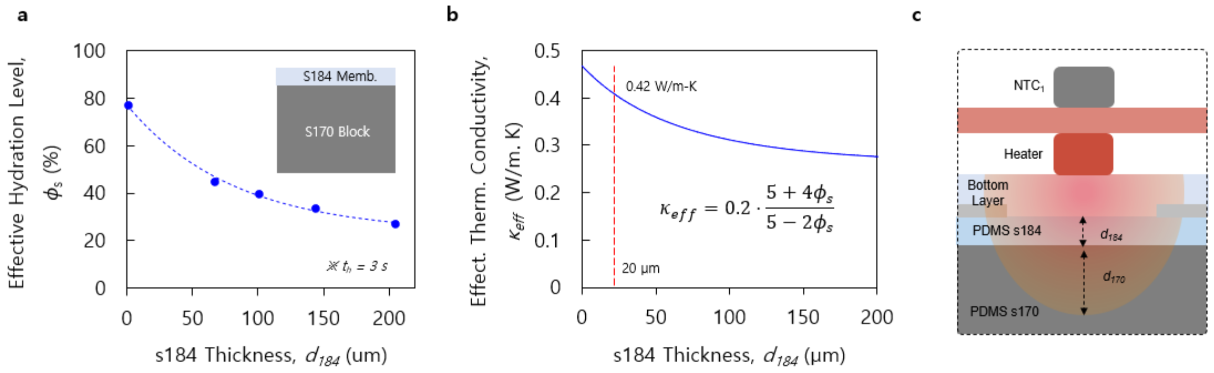

Figure S9. **Skin phantom bench-top experiment. a**. Effective hydration level measured by a kin hydration sensor ($h_{Cu} = 5\,\mu m$, $h_{Si} = 70\,\mu m$) on the skin phantom with different PDMS s184 membrane thicknesses. Measurement time is 3 s. **b**, Corresponding converted effective thermal conductivity. **c**. Schematic of the heat transfer into the skin phantom. The heat transfer reaches a quasi-steady state 3 s after the heater actuation.

Based on the assumption of quasi-steady state heat transfer, Equation R1 yields an estimate of t_{170} that corresponds to the previous estimate of the effective thermal conductivity ($\kappa_{eff} = 0.42\ W/m \cdot K$).

$$\frac{1}{\kappa_{eff}} = \frac{d_{184}/0.2 + d_{170}/0.47}{d_{184} + d_{170}} \tag{S8}$$

The resulting estimate for t_{170} is 150 μm, such that the total measurement depth including the d_{184} becomes 170 μm. Considering that the actual SC is not perfectly dry, this result suggests that the skin hydration sensor with 3 s measurement protocol could detect the skin hydration level up to about 200 μm inside the skin. As mentioned in the main text, this range covers most interesting skin structures (SC and upper epidermis).

Supplementary References

[1] a) S. R. Madhvapathy, Y. Ma, M. Patel, S. Krishnan, C. Wei, Y. Li, S. Xu, X. Feng, Y. Huang, J. A. Rogers, *Adv. Funct. Mater.* **2018**, *28* (34), 1802083, https://doi.org/10.1002/adfm.201802083; b) K.Kwon, H. Wang, J. Lim, K. S. Chun, H. Jang, I. Yoo, D. Wu, A. J. Chen, C. G. Gu, L. Lipschultz, J. U. Kim, J. Kim, H. Jeong, H. Luan, Y. Park, C.-J. Su, Y. Ishida, S. R. Madhvapathy, A. Ikoma, J. W. Kwak, D. S. Yang, A. Banks, S. Xu, Y. Huang, J.-K. Chang, J. A. Rogers, *Proc. Natl. Acad. Sci. U.S.A* **2021**, *118* (5), e2020398118, https://doi.org/10.1073/pnas.2020398118.

[2] I. H. Tavman, H. Akinci, *Int. Commun. Heat Mass Transf.* **2000**, *27* (2), 253, https://doi.org/10.1016/S0735-1933(00)00106-8.

[3] M. W. Dewhirst, B. L. Viglianti, M. Lora-Michiels, M. Hanson, P. J. Hoopes, *Int. J. Hypeth.* **2003**, *19* (3), 267, https://doi.org/10.1080/0265673031000119006.

[4] G. Swift, T. S. Molinski, W. Lehn, *IEEE Trans. Power Deliv.* **2001**, *16* (2), 171, https://doi.org/10.1109/61.915478.

[5] K. O'goshi, J. Serup, *J. Skin Res. Technol.* **2007**, *13* (1), 13, https://doi.org/10.1111/j.1600-0846.2006.00200.x.

[6] a) R. F. Furchgott, P. M. Vanhoutte, *FASEB J* **1989**, *3* (9), 2007, https://doi.org/https://doi.org/10.1096/fasebj.3.9.2545495; b) E. L. Boulpaep, in (Ed.: E. L. B. Walter F. Boron), Elsevier, Philadelphia **2016**, Ch. 20.
